# College and Hospital Notes

**Published:** 1899-05

**Authors:** 


### COLLEGE AND HOSPITAL NOTES.

The Medico-Chirurgical College petitioned the Common Pleas Court, No. 3, for leave to amend its charter so as to grant the diplomas and degrees in dental surgery, etc. This was resisted by the Philadelphia Dental College on the ground of want of authority to do so and other technical grounds. The Common Pleas Court decided in favor of the Medico-Chirurgical College and the Dental College took an appeal from this decision. The Supreme Court, in an opinion by Justice Deab, has lately confirmed the decision of the lower court and dismissed the appeal. Under this decree of the Supreme Court the college will resume its former functions and grant degrees in dental surgery.

---

DRS. JELKS AND HOLLAND, editors of the *Hot Springs Medical Journal*, opened the Ozark Sanatorium, Hot Springs, Ark., March 15th, for the reception of patients. The building is four stories high, with hydraulic elevator. It has bath tubs on each floor, and a large bath room in the basement. It has a government hot water privilege and the sanitary and hygienic arrangements of the building are perfect. It has a well-appointed surgical department, fitted up with the latest appliances, the operating room of which has been constructed with great care and has not a superior in the west.

Especial care will be taken to provide for patients who need dietetic treatment. The sanitorium building is surrounded by verandas, and has large grounds, beautifully shaded with forest trees, affording ample opportunity for fresh air and exercise. Trained nurses are in constant attendance and the institution is in charge of a competent matron.

---

THE Buffalo General Hospital has been the recent recipient of a pleasant surprise. This was a gift of $5,000 from Mrs. Isabella Higgins Moore, of Bethlehem, Pa. This money is to be invested and the interest therefrom is to be used for the maintenance of a bed to be known as the "Higgins Memorial Bed." The trustees, in accepting the gift, expressed their appreciation of Mrs. Moore's deep interest in the work of the hospital.

The hospital has also received a gift of $1,000 by the will of Milo R. Eames. A resolution was passed thanking the executors and family.

Through the efficient services of the Hon. D. S. Alexander, the hospital has secured about $1,850.00 in payment by the general government for the care, medical treatment and nursing of the sick soldiers who returned from camp last fall.

---

THE Buffalo State Hospital finds itself sadly embarrassed by the lack of proper land area. The State Lunacy Commission and the trustees of the hospital have come to the conclusion that the hospital grounds are not large enough for any additional buildings. This year only sufficient money will be set aside to make the necessary repairs and for the proper maintenance of the hospital.

Dr. A. W. Hurd, the superintendent, is frequently complimented for the record he is making at the hospital. The institution ranks amongst the highest throughout the country. During the past year the average cost for keeping each patient was only $3.34 per week, one of the lowest in the state. This average was the lowest ever attained by the hospital.

The trustees of the hospital are trying to rent a small farm in the vicinity of Buffalo. The pasturage on the state farm is very poor and the trustees believe it would be a good business investment to rent a small farm and quarter a limited number of patients on it to take care of the cows and swine.

---

THE Medical Department of the University of Buffalo announces a change in its teaching chairs to take effect at the commencement of the ensuing academic year. Until a short time ago the faculty consisted of seven members. Each member was a professor in a certain branch of medical science. One chair, which was filled by the professor of physiology, will not be represented in the faculty hereafter. At a recent meeting of the trustees of the University it was decided to divide the chair of materia medica and clinical medicine and appoint a professor for each division.

Dr. Eli Long has been elected professor of the separate chair of materia medica, while Dr. Pohlman will continue to be professor of physiology in the University. Under the new rules of the University it will not be necessary for the professor of physiology to be a member of the governing faculty.

---

SYRACUSE University has been making rapid strides during the past five years. The 1898-99 catalogue, which has just been issued, shows marvelous development in every department during the past twelve months. When the administration of Chancellor Day began five years ago there were less than 600 resident students. The present number of students in the four colleges—liberal arts, fine arts, medicine and law—is 1,152, which shows an increase of 60 over last year. No better evidence of the growth of Syracuse University is needed than the increase in size of the new catalogue. Five years ago the catalogue numbered only about 100 pages. The new catalogue contains 252 pages.

The increase in the faculties corresponds with the increase in the number of students and general growth of the university. The faculty numbers 132 members, an increase of 11 over last year. Slight changes have been made in the curriculum of the College of Medicine which includes four years' study.

